# Mortality and Body Mass Index in Burn Patients: Experience from a Tertiary Referral Burn Center in Southern Iran

**DOI:** 10.29252/wjps.8.3.382

**Published:** 2019-09

**Authors:** Abdolkhalegh Keshavarzi, Sina Kardeh, Maryam Dehghankhalili, Mohammad Hossein Varahram, Mohsen Omidi, Mitra Zardosht, Davood Mehrabani

**Affiliations:** 1 Burn and Wound Healing Research Center, Shiraz University of Medical Sciences, Shiraz, Iran;; 2 Department of Surgery, Shiraz University of Medical Sciences, Shiraz, Iran;; 3 Student Research Committee, Shiraz University of Medical Sciences, Shiraz, Iran;; 4 Cellular and Molecular Medicine Student Research Group, School of Medicine, Shiraz University of Medical Sciences, Shiraz, Iran;; 5 Stem Cell Technology Research Center, Shiraz University of Medical Sciences, Shiraz, Iran;; 6 Comparative and Experimental Medicine Center, Shiraz University of Medical Sciences, Shiraz, Iran

**Keywords:** Body Mass Index, Burn, Mortality, Obesity, Risk factor

## Abstract

**BACKGROUND:**

The role of obesity has been widely studied as a determinant factor of increasing mortality in surgical patients. In this study we aimed to investigate the association of mortality determinants with obesity classification and BMI score in burn patients admitted to a tertiary referral center in Southern Iran.

**METHODS:**

In this retrospective cross-sectional study, medical profiles of burn patients admitted from 2016 to 2017 were obtained from Amiralmomenin Burn Hospital, a tertiary referral burn center affiliated to Shiraz University of Medical Sciences, Shiraz, Iran. Demographic, and clinical characteristics as well as patient outcomes were recorded to determine prognostic factors in fatal burns based on anthropometric measurements.

**RESULTS:**

Among 101 patients who were enrolled in this study including 73 males and 28 females, mean age was 34.85±12.04 years, total burn surface area (TBSA) was 37.37 (10.50%), BMI was 25.46±5.33 kg/m^2^ and hospital stay was 22.28±13.62 days. Overall mortality rate was 24.7% with 25 expired cases. Logistic regression demonstrated significant association of older age, male gender, and greater TBSA with mortality. However, difference in mortality rate in patients with BMI of 25 kg/m2 (27.4%) in comparison to patients with BMI<25 kg/m2 (18%) did not reach statistical significance.

**CONCLUSION:**

Although patients with higher BMI had increased mortality rate following burn injury, this finding showed no significant association. Further studies with larger samples may be necessary to conclude a causal association between BMI and mortality in burn patients.

## INTRODUCTION

Burn injuries impose a great burden in developing countries.^1^^-^^3^ Despite the remarkable advances in management of burn patients, burn injuries continue to claim a high toll, particularly in predisposed patients and severe burns.^4^ The mortality rate of patients is correlated with characteristics of patients, extent of injury^5^ and the condition of care settings.^6^ A better understanding on outcome measures especially on mortality rate in severely burned patients can improve the consequences of health care and advances in surgical techniques and guidelines in burn decision making. Obesity was shown to be a determinant factor in increase of all-cause mortalities.^7^

The global epidemic of obesity enrolled our population due to alterations in eating habits and life-styles^8^ is considered as a major risk factor for the rising trend toward chronic diseases in Iran.^9^ The role of obesity has been studied in numerous medical conditions. Burn patients are also subject to multiple organ damages besides skin injury and may require intensive care. Obese patients undergoing surgical procedures are at greater risk of morbidity and mortality during post-operative period.^10^ Although a previous study revealed that obesity had negative impact on the outcome of burn patients, using BMI to determine the mortality as an outcome measure too.^11^

Several anthropometric measurements are available for quantification of obesity. Body mass index (BMI=weight [kg]/height [m]^2^) is a widely used tool for identification and classification of obesity. According to the World Health Organization (WHO) guideline, an adult individual with a BMI above 30 kg/m^2^ is considered obese.^12^ With the growing concern regarding obesity and the large scale of burn injuries in our country, we designed the present study in a retrospective approach to obtain an insight into management challenges of patients with high BMI and identify the determinants of mortality (gender, age, TBSA, hospital stay, obesity classification) in this group of patients.

## MATERIALS AND METHODS

In this retrospective cross-sectional study, medical records of patients with an admission date during a 1-year period from 2016 to 2017 in Amiralmonenin Burn Hospital, a referral tertiary burn center affiliated to Shiraz University of Medical Sciences, Shiraz, Iran were enrolled. Inclusion criteria were defined as age between 14 to 60 years and a TBSA of 20% to 60%. We excluded outpatients, repeated patients, incomplete medical records and cases with concurrent complications or history of psychological disorders and suicidal self-inflicted burns. Data were gathered according to the Helsinki declaration of bioethics and the principles of confidentiality were ensured. 

The study protocol was approved by the institutional review board (IRB) and Medical Ethics Committee of Shiraz University of Medical Sciences. After reviewing medical records of eligible burn patients, all data were recorded in data gathering forms. Demographic (age, gender), anthropometric, and clinical variables including duration of hospital stay, percentage of burn size (TBSA), and outcome (discharge form hospital versus death) were collected for each patient. TBSA as an indicator of the percentage of burn injured area was determined based on the Lund and Browder chart. 

BMI cut points were used to divide patients into four subgroups based on the WHO classification of obesity and were depicted in Table 1. Patients’ data were entered into an online database and all statistical analyses were performed using statistical package for social sciences software (version 20.00, Chicago, IL, USA). Variables were represented descriptively by frequency, mean and standard deviation as applicable. Chi Square and independent sample T Tests were used for comparison between patients with higher BMI (overweight and obese) and those with lower BMI (underweight and normal). The mortality risk factors were identified using multiple logistic regression model analysis with 95% confidence intervals (CI). A p-value less than 0.05 was considered as the statistical level of significance. 

**Table 1:** Classification of BMI

**Group**	**Classification**	**BMI (kg/m** ^2^ **) cut-off points **
1	Underweight (BMI <18.5, 5 cases) and Normal range (BMI=18.50, 24.99)	BMI<25
2	Overweight	25BMI30
3	Obese class I (moderate obesity: BMI=30.00, 34.99)	30BMI35
4	Obese class II (severe obesity: BMI=35.00, 39.99) Obese class III (morbid obesity: 40BMI, 2 cases)	35BMI

## RESULTS

Patients were predominantly male (72.2%, M/F=73/28) and ages ranged from 17 to 60 years with mean and standard deviation (SD) of 34.85±12.04 years. Average burn TBSA was 37.37 and 10.50 (20-60.50), respectively. Minimum duration of hospital stay was 1 day and reached to a maximum of 59 days (22.28±13.62). Fifty-six cases (55.4%) underwent surgery for skin grafting. Considering final outcomes, 76 cases (75.2%) were discharged and the overall mortality rate was 25 (24.7%). Of the total 101 patients in our study, 7.1 (6.9%) were underweight [BMI<18.5 (kg/m^2^)], 43.1 (42.5%) were normal-weight [BMI=18.5-24.9 (kg/m^2^)], 34.1 (33.6%) were overweight [BMI=25-29.9 (kg/m^2^)], and 17.1 (16.8%) cases were obese including 10 patients in obese class I category (moderate obesity) [BMI=30.00–34.99 (kg/m^2^)] and 5 patients in obese class II category (severe obesity) [BMI=35.00-39.99 (kg/m^2^)]. 

Only 2 patients were categorized in obese class III (morbid obesity) [BMI=40.00 (kg/m^2^)]. The mean and SD of BMI for the whole group was 25.46±5.33 kg/m^2^. Table 2 shows a summary of demographical, clinical characteristics and final outcomes with burn mortality risk factor variables for the entire cohort of 101 patients categorized in different BMI classes comparing normal cases with those who had a BMI greater than the WHO cut points for obesity definition. We used a multivariate logistic regression model analysis to determine the risk factors of mortality in our series. 

**Table 2:** Demographical, burn and mortality data of the study cohort as well as subgroups defined by the BMI cut points

	**BMI<25**	**BMI=25–30**	**BMI=30–35**	**35BMI**	**All**
Number of patients	50	34	10	7	101
Male/female	35/15	26/8	6/4	6/1	73/28
Age in years	33.36±11.64 17-59	36.97±12.94 18-60	36.50±8.36 24-58	28.00±7.18 21-41	34.85±12.04 17-60
TBSA	35.29±9.67 20-60.5	38.71±11.48 21-59.50	37.55±11.04 24-60	40.42±8.88 26-52	37.37±10.50 20-60.5
Skin grafting	27 (54%)	19 (55.9%)	8 (80%)	2 (28.6%)	56 (55.4%)
Mortality	9 (18%)	8 (23.5%)	5 (50%)	1 (14.3%)	25 (24.7%)
BMI	21.39±2.42 14.20-24.9	27.14±1.34 25-29.30	31.44±0.86 30.40-33.50	37.88±4.71 35-47	25.46±5.33 14.20-47.70
Hospital stay - days	23.51±13.99 1-58	20.70±13.53 1-59	22.30±12.79 3-43	18.85±11.75 4-42	22.28±13.62 1-59

As shown in Table 3, gender, age, and burn size determined by TBSA% were found to be significantly associated with mortality while there was no significant correlation between increased mortality with BMI. Based on gender, average BMI was higher in male patients (25.7±5.5 kg/m^2^) in comparison to the female patients (24.9±4.7 kg/m^2^); however, this difference was not statistically significant. Comparison between patients with BMI less than 25 kg/m^2^ (normal and underweight, mortality=9, 18%) and those with BMI greater or equal to 25 kg/m^2^ (obese and overweight, mortality=14, 27.4%) showed that observed mortality was not significantly higher when patients had BMI greater or equal to 25 kg/m^2^ (*p*>0.05). 

**Table 3:** Logistic regression analysis of effect of patients’ and injury characteristics on mortality in patients with severe burns

**Variable**	***p*** ** value**	**95% CI lower**	**95% CI upper**
Age	0.04	0.84	0.99
Sex	0.00	0.68	0.91
TBSA	0.00	0.81	0.96
Hospital Stay	0.61	0.93	1.04
Skin graft	0.65	0.28	7.34
BMI	0.20	0.77	1.05

Considering other mortality risk factors, the mean and SD of TBSA was 35.29 (20-60.50) in cases with BMI less than 25 kg/m^2^ and 38.72 (21-60) in cases with BMI greater or equal to 25 kg/m^2^. Nevertheless, this increase in TBSA was not found to be statistically significant (*p*>0.05). Furthermore, difference in average hospital stay between these 2 groups failed to reach statistical significance (*p*>0.05). 

## DISCUSSION

Survival and mortality following burns are influenced by several factors, among them TBSA of burn and patient’s age are the most prominent predictors of outcome.^13^ Since the formation of the original concept of BMI, which is now a universally accepted measure to define and classify obesity, it has always been closely associated with statistics of differential mortality rates implying a distinct increase in risks of disability and mortality at levels above its normal cut point.^14^^,^^15^

In spite of continuous improvement observed in survival of severely burned patients over the last six decades, reports on outcome analytics of patients with different forms of obesity have been scarce. Moreover, obesity was introduced as a contributing factor in morbidity and mortality of burn patients, almost three decades ago and most of the earlier studies on burn and obesity have not classified results based on BMI cut points to determine the shift in risk of mortality.^16^^,^^17^ Our study supports other studies indicating that obesity may affect burn outcome as death rate of patients with BMI=25 (kg/m^2^) to be higher in comparison to patients with BMI<25 kg/m^2^, but the difference was not significant. The first study on this context was conducted in 1990 at Parkland Memorial Burn Hospital categorizing patients based on ideal body weight or total weight and not using BMI, highlighted an increased risk of mortality in obese burn patients, especially when patients’ weight was above 120 kg.^18^

In 1992, an investigation by the United States Army Institute of Surgical Research on 7 morbidly obese patients with a mean BMI of 50.8 (kg/m^2^) showed a higher mortality than expected mortality (57%) and an increased incidence of pulmonary embolism.^19^ In 2008, a study from the Burns Centre at Washington Hospital by Carpenter* et al.* assessed a database of 101,450 cases in the national burn database and reported that obese patients were 4.1 times more likely to stay at hospital for more than a week and 2.6 times more likely to die compared with patients who were not described as obese.^20^

In 2011, a study which was retrospective including 95 patients treated over 2-year period in a dedicated burn unit revealed a BMI of 35, a tilt point associated with a higher mortality than predicted mortality, when compared to burned patients with a normal BMI.^21^ On the other hand, assessing the impact of obesity on morbidity and mortality in a total of 405 patients with more than 20% TBSA burn requiring at least 1 surgical intervention, demonstrated no differences in primary and secondary outcomes; when normal weight patients were compared with obese patients. However, stratifying adult patients in obesity categories, showed that despite improved survival in patients with mild obesity, morbidly obese patients had the highest mortality.^22^

Due to easy and simple retrieval, observed mortality remains the most common measure for outcome evaluation following burn injury; however, optimal using of mortality models for clinical decision making are limited as their estimation is dependent on a diverse set of admission parameters which are heterogeneous in nature and also among different similar studies. Due to incompleteness of data in this retrospective evaluation, we could not identify comorbidities and their impact on mortality. 

However, as care is often challenging and compromised due to the physical constraints of these patients, complications are disproportionate to burn size, location, and age.^18^ In 1993, a study of burn obese patients requiring ITU care denoted to specific complications including greater incidence of infection, higher resting energy expenditure measurements and prolonged need for antibiotic therapy. Moreover, data demonstrated prolonged catabolic phase and delayed anabolic metabolism marked by aberration in level of biochemicals such as insulin, and glucagon.^23^

In addition, evaluating the relationship between obesity and functional outcomes in a group of 221 burn patients, Farrell *et al.* indicated that higher BMI may not only decrease the likelihood of post-hospitalization discharge, but also can contribute to lower functional independence measure scores.^24^ Overall, the higher morbidity and mortality rate in obese patients can be attributed to 2 main factors of (i) multiple co-existing and related health disorders that increased the risk of developing all known organ and functional systems diseases, and (ii) higher complications of surgery.^25^

Considering the increasing rate of obesity in Iranian adult population, recently estimated about 21.7%, these patients need to be referred to a burn unit adequately prepared with the equipment and expertise to manage obese patients as they are presented with major problems for the burn team, especially in the areas of wound and general nursing care.^8^^,^^26^ Despite the limitations of this study by relatively small sample size and its retrospective method, it provides further insight into the global obesity epidemic in general and its impact on the cohort of patients sustaining large burns. This observation is not only important to minimize the mortality and complications of burns by using optimized burn care given by a specialist team for obese patients, but also to draw attention of strategic public health authorities involved in planning the prevention, management, rehabilitation, and prognosis of burns in this emerging subpopulation of patients.
